# Examining the dynamics of the relationship between water pH and other water quality parameters in ground and surface water systems

**DOI:** 10.1371/journal.pone.0262117

**Published:** 2022-01-25

**Authors:** Benjamin M. Saalidong, Simon Appah Aram, Samuel Otu, Patrick Osei Lartey

**Affiliations:** 1 Department of Geosciences, Taiyuan University of Technology, Taiyuan, People’s Republic of China; 2 College of Safety and Emergency Management Engineering, Taiyuan University of Technology, Taiyuan, People’s Republic of China; 3 Department of Earth and Environmental Science, New Mexico Institute of Mining and Technology, Socorro, NM, United States of America; 4 Ministry of Education Key Laboratory of Interface and Engineering in Advanced Materials, Research Center of Advanced Materials Science and Technology, Taiyuan University of Technology, Taiyuan, People’s Republic of China; Bangabandhu Sheikh Mujibur Rahman Agricultural University, BANGLADESH

## Abstract

This study evaluated the relationship between water pH and the physicochemical properties of water while controlling for the influence of heavy metals and bacteriological factors using a nested logistic regression model. The study further sought to assess how these relationships are compared across confined water systems (ground water) and open water systems (surface water). Samples were collected from 100 groundwater and 132 surface water locations in the Tarkwa mining area. For the zero-order relationship in groundwater, EC, TDS, TSS, Ca, SO_4_^2-^, total alkalinity, Zn, Mn, Cu, faecal and total coliform were more likely to predict optimal water pH. For surface water however, only TSS, turbidity, total alkalinity and Ca were significant predictors of optimal pH levels. At the multivariate level for groundwater, TDS, turbidity, total alkalinity and TSS were more likely to predict optimal water pH while EC, Mg, Mn and Zn were associated with non-optimal water pH. For the surface water system, turbidity, Ca, TSS, NO_3_, Mn and total coliform were associated with optimal water pH while SO_4_^2-^, EC, Zn, Cu, and faecal coliform were associated with non-optimal water pH. The non-robustness of predictors in the surface water models were conspicuous. The results indicate that the relationship between water pH and other water quality parameters are different in different water systems and can be influenced by the presence of other parameters. Associations between parameters are steadier in groundwater systems due to its confined nature. Extraneous inputs and physical variations subject surface water to constant variations which reflected in the non-robustness of the predictors. However, the carbonate system was influential in how water quality parameters associate with one another in both ground and surface water systems. This study affirms that chemical constituents in natural water bodies react in the environment in far more complicated ways than if they were isolated and that the interaction between various parameters could predict the quality of water in a particular system.

## Introduction

Water is and will continue to be an important part of life. water bodies such as lakes, rivers and streams are the most essential reservoirs for freshwater [1]. Groundwater remains an essential source of potable water, serving as the primary water resource in arid regions. Compromising the quality of ground and surface water endangers the health and safety of residents within its catchment areas. Assessing the quality of water is mainly based on its physicochemical components, biological quality and heavy metals concentrations [2]. Water systems are considered contaminated when the presence of organic, inorganic, biological, thermal or radiological substances in them are at a level which tend to degrade or adversely affect the quality of water and consequently affecting it usefulness [3].

The quality of water in a reservoir is governed by anthropogenic processes such as industrial, agricultural, human exploitations and natural process including precipitation, weathering, erosions, mineral deposits and other geological phenomena [4]. Surface waters are the most susceptible and vulnerable water bodies to contamination as a result of being exposed to various types of waste and runoffs [5]. Ground water on the other hand is better protected against direct runoffs and waste disposals, however, once contaminated, it remains contaminated for longer periods [6], and as such there is the need to keep it safe for use.

pH is probably by far the most important physicochemical parameter controlling the behavior of other water quality parameters as well as metals concentration in the aquatic environments [7]. Chemical processes in aquatic systems such as acid-base reactions, solubility reactions, oxidation-reduction reactions and complexations are all influenced by hydrogen ions concentration (pH). Water bodies around the vicinity of mining activities are susceptible to receiving metals from dumpsite leachate and other waste discharge from the mining activities [8]. Metal pollution has become a major concern due to their ability to bioaccumulate along the food chain [9]. The availability of these metals can however be influenced by pH, making pH an important factor in determining the chemical and biological properties of water.

pH may also influence the lives of bacteria and the availability of other contaminants in water. In general, very high or very low pH can make water unpleasant for certain purposes. At very high pH, metals tend to precipitate while chemicals such as ammonia become toxic to aquatic life; water tend to have unpleasant smell and taste in alkaline conditions [10]. At low pH, solubility of metals tend to be high, chemicals like cyanide and sulphide become more toxic. Acidic waters also corrode metal pipes. Therefore, heavy metals in water with a low pH tend to be more toxic, as they become more soluble and bioavailable. Exposures to extreme water pH via drinking and skin contact are known to be associated with irritation to the eyes, skin, and mucous membranes [11]. Many municipal water suppliers voluntarily test the pH of their water to monitor for pollutants [12]. Thus, the determination of pH could serve as a sensitive indicator for contamination.

Water quality monitoring is given a high priority for the determination of current conditions and long-term trends for effective management. Given that water is one of the most important life requirements and taking into account the challenges of its quality management, there is the need to identify and assess the sources of contamination through monitoring and evaluation. However, high cost of data sampling and collection provides a challenge on the implementation of water quality monitoring programs. Field measurements may not always give a perfect view of the reality due to sensors having bad contact resulting from fouling, clogging or lack of maintenance. Measurement can also be influenced by external factors: humidity, temperature extremes or electromagnetic fields. The calibration of the measuring instrument may also give rise to problems. In an attempt to reduce the challenges in measurement and monitoring, water quality modelling provides an alternative for characterizing and predicting water quality parameters and to evaluate potential contamination using few measured parameters.

Several modelling techniques has been deployed in water quality monitoring and evaluation including cluster analysis (CA), principal component analysis (PCA), factor analysis (FA), Stepwise logistic regression and multiple linear regression (MLR). Given the large amount of data for assessing quality parameters, there is the need to develop indirect approaches (models) to predicts fluctuation in the factors affecting the quality of the environment. Multidisciplinary research techniques provide opportunities in addressing the challenges associated with understanding the links that exist between mining operations and how it affects the environment. These models offer an alternative approach to a better interpretation of data and to understand water quality [13–15], while making it possible to assess factors influencing the behavior of an environmental system and offers a valuable tool for managing resources as well as solution to pollution problems.

Models such as PCA provides understanding to the underlying relationships between the variables. Verma and Singh [16] successfully used an artificial neural network (ANN) model to predict water quality parameters of coalmine discharge, Individual techniques such as multiple linear regression (MLR) might not be very useful in addressing problems involving complex and non-linear data and thus might not provide the best and accurate prediction [17], it is also difficult to describe the quality of water in a quantitative manner by relying solely on models. However, methods that combines these models will allow a more accurate prediction. The task of monitoring water quality can be facilitated if the relationship between various water quality parameters can be established, the inter-parameter relationship offers remarkable information on the source and pathway of parameters. The existence of such associations can help predict the existence of other parameters. Knowledge of these relationships can also help assess conditions of unmonitored water bodies by inferring from already measured parameters and also identify human activities that significantly contribute to pollution as well as areas that are at risk and promote management practices to reduce non-point source pollution [18].

In this study, a nested logistic regression model was used to examine the relationship between water pH levels and physicochemical factors while controlling for heavy metals and biological factors in water systems (surface and ground water) in the Tarkwa mining area. Modelled and predicted pH could serve as means of detecting abnormal values, discontinuities and recording drifts from routine measurements. In as much as pH affects the biological, physical and chemical properties of water, it is also affected by the water’s geochemistry. Ewusi et al. [13] affirmed in their study that regression models were appropriate for water quality modelling.

## Materials and methods

### Study area

Samples for this study were taken in Tarkwa, a mining town in the Western Region of Ghana. Tarkwa lies within the south-western equatorial climate zone. The country falls between latitudes 4° 0ʹ 0″ N and 5° 40ʹ 0″ N and longitudes 1° 45ʹ 0″ W and 2° 1ʹ 0″ W. A total of 232 locations (ground water = 100 and surface water = 132) were sampled for this study. Sampling was done on quarterly basis between January 2019 and December 2019. Tarkwa is one of the areas in the country that experience high rainfall. This causes heavy runoffs and leaching of surface soil chemicals. The area was selected for this study due to the high-level of anthropogenic activities including mining activities, welding and other mechanical servicing activities that serve as the main sources of pollution to water supply systems [19, 20].

Boreholes, hand-dug wells, streams and rivers are the major source of water supply in Tarkwa for both domestic and commercial purpose. The majority of these water supply systems serve as a source of drinking water for nearby communities. The average well depth in the area is about 35.4m [21]. The quality of the water supply systems in the area is highly affected by mine contaminants and mining-related activities, leakage from underground storage tanks, improper waste disposal and agrochemicals from agricultural fields. The study area is located within a drainage basin of the Ankobra River Basin. The Bonsa, Huni and Ankobra Rivers and their tributaries are the main sources of drainage system in the area [21, 22].

### Data description

A total of 17 parameters, which include 10 physicochemical parameters (electrical conductivity, total dissolved solids, total suspended solids, turbidity, total alkalinity, magnesium, calcium, sulphate, nitrate and phosphate), 5 heavy metals (arsenic, zinc, iron, manganese and copper) and 2 biological parameters (faecal coliform and total coliform) were obtained from ground and surface water systems in the study area. These parameters were carefully chosen based on their data availability, significance and concentrations with respect to the WHO guideline values.

Water pH was the focus variable of this research. pH was selected as the response variable because pH is notably one of the most important physicochemical parameters that controls the behavior of other water quality parameters as well as metals concentration in aquatic environments [7]. Chemical processes in aquatic systems such as acid-base reactions, solubility reactions, oxidation-reduction reactions and complexation are all influenced by pH. The WHO has a standard drinking water guideline for pH. In this instance, all drinking waters should be within a pH range of 6.5–8.5. In this study, pH values that were within this range were classified as optimal and coded with “1” while pH values that were outside of this standard were classified as “non-optimal” and coded as “0” to get a binary outcome (non-optimal/ optimal).

### Logistic regression analysis

In this study, a logistic regression statistical model was deployed, This model relates to the response variable through a link function by allowing the magnitude of the variance of each measurement to be a function of its predicted value under the assumption of binary response (non-optimal/ optimal) [23], Via the link function, there are several potential techniques that could be deployed for a logistic regression analysis: the logit model, probit model, negative log-log and complementary log-log model. Both logit and probit link functions have the same property, that is the probability that an observation in a specified category of a binary outcome variable has the same probability of approaching 0 as well as approaching 1 (50% non-optimal, 50% optimal). Given that, the observations of a binary outcome have an asymmetrical success of probability, that is, fewer 0s than 1s or more 0s than 1s, then the link function complementary log-log or negative log-log is chosen respectively. In this study 64% and 79.5% of the locations had optimal pH for drinking water for ground water and surface water respectively. For this reason, the complementary log-log link function was appropriate for modelling water pH levels.

The odds ratios (OR) were built in a nested model starting from the physicochemical model, heavy metals model and bacteriological model. An OR of 1 meant that higher values of the predictor did not affect the odds of optimum or non-optimum water pH; OR > 1 meant that the predictor was associated with odds of optimum water pH; and OR < 1 meant the predictor variable was associated with odds of non-optimum water pH.

All statistical analyses were performed using Stata 15 (StataCorp, College Station, Texas) SE software at a statistical significance of 0.05 and at a confidence interval of 95%.

### Water sampling

The sampling was carried out in accordance with the protocols developed by the America Public Health Association (APHA) [24]. Sampling bottles were washed with detergent and rinsed with 10% hydrochloric acid and double-distilled water prior to sampling. At each of the sampling locations, bottles used to collect samples were thoroughly rinsed with the water to be sampled three times to reduce possible contamination of the sampling bottles. Surface water samples were taken midstream with conscious effort not to disturb water sediments by gently submerging the sample bottle horizontally into the water to fill the bottles while facing upstream, taking reasonable measures to avoid suspended/floating debris. Thus, surface water samples were collected at the subsurface in order to avoid the colloidal layer as this can influence the concentration of certain parameters. Personnel entry into the water body was minimized as much as possible. 1000 mL of water was collected from each sample location using two 500 mL transparent plastic bottles, which were placed in an opaque material (black polyethylene bag), tied and finally kept in a cooler box. Bottles containing samples were labelled using first letters of sampling site and numbers. This procedure minimized the possible growth of micro bacteria, flocculation and reduce any adsorption on container surfaces, processes which could affect the results.

Water from the community boreholes was collected at the faucet after it had been pumped for a while to obtain a steady flow before sampling. This was to be sure that the water being collected is freshly extracted from the borehole.

### Field analysis

pH, conductivity and turbidity were measured *in situ* during the sampling. Calibrations were conducted in the field at the sample site. The pH probe was calibrated with pH 7 and 10 buffer solutions on the day of sampling.

### Laboratory analysis

Laboratory tests were conducted in compliance with “Standard Methods for the Examination of Water and Wastewater” of the American Public Health Association, 1998 Edition. Analysis of metals As, Fe, Mn, Cu and Zn were carried out by homogenizing samples, filtered and acid-digested in accordance with USEPA protocol 2002 and analyzed using flame atomic absorption spectroscopy (AA240FS) following USEPA protocol 2002 [25]. Unprocessed water samples were also analyzed for electrical conductivity, and for chloride, sulphate, nitrate, phosphate, and alkalinity concentrations. Faecal coliform and total coliform were also determined by the membrane filtration technique.

## Results

### Descriptive statistics

Table 1 shows a statistical summary (mean, standard deviation, minimum and maximum) of pH and the predicting variables selected for the study. A total of 100 ground water locations were sampled. Maximum and minimum pH values recorded were 7.850 and 5.240 respectively with a mean pH of 6.737, indicating an acidic to slightly alkaline groundwater samples. Out of the 100 locations, 36 recorded pH values outside the range of the WHO standard for drinking water [26]. The remaining 64 locations however recorded pH values within the WHO guidelines for drinking water quality.

**Table 1:** Statistical summary of predictors and explanatory variables.

Variables	Ground water				Surface water			
	Mean	Std. Dev.	Min	Max	Mean	Std. Dev.	Min	Max
**pH**	6.737	0.663	5.240	7.85	7.005	0.82	4.16	9.95
**Conductivity**	281.37	168.218	35	751	304.439	182.904	39	821
**Total dissolve solids**	185.03	118.735	14	682	199.508	124.457	14	542
**Total suspended solids**	72.7	202.399	1	1310	190.682	894.586	3	7300
**Turbidity**	61.346	220.107	0.01	1540	89.737	307.706	0.13	3180
**Magnesium**	5.788	6.17	0.01	40.3	6.74	5.126	0.01	31
**Calcium**	21.74	27.268	0.01	200	25.364	21.231	0.01	100
**Nitrate**	9.53	15.545	0	86.2	23.904	26.122	0.01	118
**Sulphate**	28.386	46.813	0	401	58.616	73.46	0	338
**Phosphate**	5.825	10.278	0.05	39.7	7.522	11.972	0.1	55.2
**Arsenic**	0.005	0.005	0.001	0.029	0.005	0.006	0.001	0.025
**Zinc**	0.131	0.267	0.01	2.14	0.102	0.165	0.01	0.8
**Iron**	0.056	0.14	0.01	1.3	0.129	0.232	0.01	1.5
**Manganese**	0.017	0.020	0.001	0.098	0.01	0.024	0.001	0.25
**Copper**	0.567	0.855	0	2.95	0.386	0.753	0	5
**Total alkalinity**	0	1	-1.02	5.87	0	1	-0.825	9.272
**Faecal coliform**	207.61	1159.458	0	8220	14.288	106.596	0	870
**Total coliform**	1461.08	5971.452	0	41100	871.174	4452.74	0	35620

For surface water sources, there were132 sample locations. The mean pH value recorded was 7.005 With maximum and minimum values of 9.950 and 4.160 respectively. Out of the 132 locations, 27 recorded pH values outside the range of the WHO standard for drinking water quality.

Most of the other physicochemical parameters were within the guideline limits with few sample locations recording values above the guideline limit. On average, surface water recorded high values for heavy metals as compared to groundwater. Coliform bacteria were relatively high in ground water than surface water sources.

### Correlation analysis for water quality parameters in groundwater and surface water sources

Pearson’s correlation analysis (r) for the selected parameters were carried out. From the correlation matrix for ground water (Table 2), conductivity was highly correlated with total dissolved solids (r = 0.963). The strong correlation between conductivity and total dissolved solids gives an indication of the extent to which salts dissociate into ions and influence conductivity. Conductivity is the ability of water to conduct electrical current and it is related to the concentration of ionized substances in the water. Total suspended solids was moderately correlated with magnesium (r = 0.601), calcium (r = 0.556) and sulphate (r = 0.682). Magnesium showed a moderate correlation with total alkalinity (r = 0.633), while phosphate was also moderately correlated with total coliform (r = -0.525). pH was moderately correlated with nitrate (r = -0.525). For surface water sources in Table 3, conductivity again showed a strong correlation with total dissolved solids (r = 0.963) and moderately correlated with nitrate (r = 0.581). pH was weakly correlated with all parameters selected for the study.

**Table 2:** Pearson’s correlation matrix for analyzed water quality parameters in groundwater.

	Ground water Correlation																	
	pH	EC	TDS	TSS	Turbidity	Mg	Ca	NO_3_	SO_4_^2-^	PO_4_^3-^	AS	Zn	Fe	Mn	Cu	T.alkalinity	F. coli	T. coli
**pH**	1.000																	
**EC**	0.381	1.000																
**TDS**	0.354	**0.963**	1.000															
**TSS**	0.046	0.135	0.115	1.000														
**Turbidity**	-0.341	-0.140	-0.105	0.325	1.000													
**Mg**	-0.066	0.328	0.408	**0.601**	0.394	1.000												
**Ca**	0.428	0.457	0.445	**0.556**	-0.053	0.582	1.000											
**NO**_**3**_	**-0.525**	**-0.253**	-0.210	0.195	0.321	0.225	-0.179	1.000										
**SO**_**4**_^**2-**^	-0.011	0.181	0.152	**0.682**	0.247	0.177	0.080	0.288	1.000									
**PO**_**4**_^**3-**^	-0.050	0.248	0.366	0.253	0.183	0.455	0.040	0.224	0.282	1.000								
**As**	0.004	0.026	-0.005	-0.187	-0.319	-0.135	0.041	-0.193	-0.237	-0.272	1.000							
**Zn**	-0.359	-0.194	-0.029	-0.085	0.206	0.169	-0.356	0.325	-0.027	0.382	-0.107	1.000						
**Fe**	0.088	-0.094	-0.014	0.173	0.388	0.269	0.027	0.070	0.122	0.188	-0.226	0.222	1.000					
**Mn**	-0.272	-0.174	-0.118	-0.013	0.365	0.033	-0.166	0.338	0.059	0.165	-0.135	0.219	0.231	1.000				
**Cu**	-0.280	-0.174	-0.210	-0.072	0.288	-0.033	-0.273	0.314	0.055	-0.269	-0.011	0.060	-0.253	0.274	1.000			
**T.alkalinity**	0.359	0.423	0.388	0.414	0.058	**0.633**	0.787	-0.148	-0.043	0.144	0.067	-0.315	0.186	0.039	-0.159	1.000		
**F. coli**	0.057	0.094	0.065	-0.061	-0.352	-0.080	0.055	0.101	-0.075	-0.141	0.078	0.051	-0.181	-0.120	0.014	0.041	1.000	
**T. Coli**	0.067	0.036	-0.036	-0.031	-0.063	-0.169	0.019	-0.219	0.052	**-0.525**	0.004	-0.212	-0.176	-0.091	0.292	-0.017	0.408	1.000

Note: *In bold are significant correlations.

**Table 3:** Pearson’s correlation matrix for analyzed water quality parameters in surface water.

	Surface water Correlation																	
	pH	EC	TDS	TSS	Turbidity	Mg	Ca	NO_3_	SO_4_^2-^	PO_4_^3-^	AS	Zn	Fe	Mn	Cu	T. alkalinity	F. coli	T. coli
**pH**	1.000																	
**Cond**	0.203	1.000																
**TDS**	0.165	**0.963**	1.000															
**TSS**	0.227	0.063	0.049	1.000														
**Turbidity**	0.278	0.090	0.059	0.692	1.000													
**Mg**	-0.008	0.276	0.329	0.394	0.294	1.000												
**Ca**	0.131	0.304	0.231	0.004	-0.051	0.130	1.000											
**No**_**3**_	0.166	**0.581**	0.542	0.127	0.070	0.336	0.268	1.000										
**SO**_**4**_^**2-**^	0.053	0.413	0.479	0.022	0.119	0.299	0.178	0.253	1.000									
**PO**_**4**_^**3-**^	0.018	0.107	0.237	0.059	0.055	0.386	-0.037	-0.035	0.361	1.000								
**As**	0.046	-0.016	-0.100	-0.020	0.119	-0.222	-0.146	-0.158	-0.062	-0.093	1.000							
**Zn**	-0.215	0.073	0.120	-0.019	-0.070	0.107	-0.129	-0.080	0.237	0.232	-0.090	1.000						
**Fe**	0.009	-0.164	-0.129	0.224	0.489	0.148	-0.212	-0.271	0.147	0.386	0.118	0.186	1.000					
**Mn**	-0.123	0.010	0.024	0.212	0.299	0.363	-0.310	0.014	0.035	0.170	0.203	0.161	0.265	1.000				
**Cu**	-0.046	-0.169	-0.158	0.240	0.124	0.004	-0.151	-0.349	-0.076	-0.141	0.033	0.187	0.102	-0.079	1.000			
**T. alkalinity**	0.306	0.343	0.274	0.382	0.354	-0.042	0.282	0.085	-0.065	-0.165	0.089	-0.196	-0.018	-0.084	0.052	1.000		
**F. coli**	0.318	-0.002	-0.033	0.059	0.075	-0.245	-0.240	0.004	-0.022	-0.254	0.142	-0.059	-0.101	-0.127	0.161	-0.029	1.000	
**T. coli**	0.369	-0.010	-0.082	-0.007	0.063	-0.313	0.092	-0.028	0.019	-0.036	0.211	-0.220	0.037	-0.228	0.032	0.290	0.223	1.000

Note: * In bold are significant correlations.

### Zero-order relationship between pH and selected water quality parameters

Table 4 shows the results of the association between individual parameters and their odds of predicting pH levels in ground and surface water sources. For ground water physicochemical factors, conductivity (OR = 1.004, p < 0.001), total dissolved solids (OR = 1.005, p < 0.001), total suspended solids (OR = 1.003, p < 0.05), calcium (OR = 1.023, p < 0.05) and sulphate (OR = 1.009, p < 0.05) were significantly associated with higher odds of optimum water pH. Similarly, higher values of total alkalinity (OR = 1.720, p < 0.05) was significantly associated with higher odds of optimum water pH. Turbidity, magnesium, nitrate and phosphate showed no association with pH in groundwater. Among the heavy metals, zinc (OR = 0.091, p < 0.05), manganese (OR = 1.37E-13, p < 0.05) and copper (OR = 0.578, p < 0.05) were statistically significant in predicting water pH levels. Here, higher values of zinc, manganese and copper were associated with non-optimal water pH. Of the bacteriological factors, faecal coliform (OR = 1.000, p < 0.05) showed significant association with pH levels, however, odds of faecal coliform did not affect the odds of predicting pH levels in ground water.

**Table 4:** Zero-order complementary log–log regression showing the relationship between pH and selected water quality parameters.

Variables	GROUND WATER					SURFACE WATER				
	OR	Robust SE	P Value	Conf. Interval		OR	Robust SE	P Value	Conf. Interval	
**Conductivity**	1.004	0.001	**0.001**	1.002	1.006	1.000	0.001	0.947	0.999	1.001
**Total dissolved solids**	1.005	0.002	**0.001**	1.002	1.008	1.000	0.001	0.771	0.999	1.002
**Total suspended solids**	1.003	0.001	**0.026**	1.000	1.006	1.008	0.004	**0.026**	1.001	1.016
**Turbidity**	1.002	0.002	0.218	0.999	1.005	1.014	0.006	**0.025**	1.002	1.027
**Total alkalinity**	1.720	0.331	**0.005**	1.179	2.509	2.014	0.573	**0.014**	1.154	3.517
**Magnesium**	1.016	0.021	0.440	0.976	1.057	1.006	0.021	0.776	0.965	1.049
**Calcium**	1.023	0.009	**0.011**	1.005	1.041	1.023	0.006	**0.000**	1.010	1.035
**Sulphate**	1.009	0.004	**0.043**	1.000	1.017	1.000	0.001	0.792	0.997	1.002
**Nitrate**	0.972	0.022	0.207	0.929	1.016	1.008	0.005	0.062	1.000	1.017
**Phosphate**	1.001	0.012	0.911	0.977	1.026	1.012	0.009	0.177	0.994	1.031
**Arsenic**	339169.8	8573454	0.614	1.03E-16	1.1E+27	1.6E-05	0.00032	0.580	1.69E-22	1.5E+12
**Zinc**	0.091	0.096	**0.023**	0.012	0.721	0.326	0.238	0.124	0.078	1.359
**Iron**	18.655	38.183	0.153	0.338	1030.359	1.445	0.753	0.479	0.521	4.010
**Manganese**	1.37E-13	1.69E-12	**0.016**	4.55E-24	0.004	0.005	0.048	0.607	5.31E-12	3871298
**Copper**	0.578	0.105	**0.003**	0.404	0.826	0.819	0.136	0.230	0.592	1.134
**Faecal coliform**	1.000	5.46E-05	**0.027**	1.000	1.000	0.991	0.016	0.570	0.961	1.022
**Total coliform**	1.000	0.000049	0.300	1.000	1.000	1.000	0.00031	0.190	1.000	1.001

Note: *In bold are significant predictors.

For surface water sources, only total suspended solids (OR = 1.008, p < 0.05), turbidity (OR = 1.014, p < 0.05), calcium (OR = 1.023, p < 0.05) and total alkalinity (OR = 2.014, p < 0.001) were statistically associated with predicting pH levels. Here, higher values of total suspended solids, turbidity, calcium and total alkalinity were associated with higher odds of optimum water pH. None of the heavy metals and bacteriological factors was significant in predicting pH levels in surface water at the bivariate level.

### Multivariate complementary log–log regression model showing the relationship between pH and selected water quality parameters for groundwater

Table 5 shows the results of the multivariate regression analysis of three different models for ground water. Model 1 presents the results for the physicochemical parameters. Model 2 accounted for physicochemical and heavy metals and in the third model, physicochemical factors together with heavy metals and biological parameters were accounted for. Model 1 showed that, conductivity (OR = 0.990, p < 0.05) and magnesium (OR = 0.735, p < 0.05) were less likely to be associated with optimal water pH. Total dissolved solids (OR = 1.020, p < 0.05), turbidity (OR = 1.002, p < 0.05) and total alkalinity (OR = 2. 780, p < 0.05) were statistically associated with higher odds of pH, thus higher values of total dissolved solids, turbidity and total alkalinity were associated with higher odds of predicting optimal water pH for drinking groundwater sources.

**Table 5:** Multivariate complementary log–log regression model predicting the relationship between pH and water quality parameters for groundwater.

	GROUND WATER														
	Physicochemical parameters					Physicochemical parameters and Heavy Metals					Physicochemical, Heavy metal and Biological parameters				
	Model 1					Model 2					Model 3				
Variables	OR	Robust SE	P Value	Conf. Interval		OR	Robust SE	P Value	Conf. Interval		OR	Robust SE	P Value	Conf. Interval	
conductivity	0.990	0.004	**0.010**	0.982	0.998	0.984	0.004	**0.000**	0.976	0.992	0.984	0.004	**0.000**	0.976	0.992
Total dissolved solids	1.020	0.007	**0.003**	1.007	1.033	1.032	0.008	**0.000**	1.016	1.048	1.032	0.008	**0.000**	1.016	1.048
Total suspended solids	1.007	0.004	0.095	0.999	1.016	1.015	0.006	**0.009**	1.004	1.027	1.016	0.006	**0.005**	1.005	1.027
Turbidity	1.002	0.001	**0.007**	1.001	1.004	1.002	0.001	**0.001**	1.001	1.003	1.002	0.001	**0.001**	1.001	1.003
Magnesium	0.735	0.073	**0.002**	0.606	0.892	0.710	0.076	**0.001**	0.576	0.876	0.709	0.074	**0.001**	0.578	0.870
Calcium	1.041	0.022	0.052	1.000	1.085	1.033	0.024	0.164	0.987	1.081	1.034	0.023	0.126	0.991	1.080
Sulphate	1.007	0.008	0.385	0.992	1.022	0.998	0.011	0.876	0.977	1.020	0.999	0.011	0.931	0.978	1.021
Nitrate	0.980	0.019	0.306	0.944	1.018	0.985	0.015	0.299	0.957	1.014	0.985	0.015	0.336	0.956	1.016
Phosphate	0.992	0.028	0.773	0.937	1.049	0.995	0.022	0.817	0.952	1.039	0.998	0.024	0.944	0.952	1.047
Total alkalinity	2.780	1.024	**0.005**	1.351	5.721	3.558	1.418	**0.001**	1.629	7.769	3.436	1.382	**0.002**	1.562	7.557
Arsenic						7.65*10^−40^	9.95E-38	0.489	1.3E-150	4.4E+71	1E-35	1.4E-33	0.557	1.4E-152	6.95E+81
Zinc						0.108	0.105	**0.022**	0.016	0.727	0.102	0.102	**0.022**	0.014	0.721
Iron						0.287	0.314	0.255	0.033	2.459	0.288	0.309	0.247	0.035	2.363
Manganese						5.71*10^−22^	1.23E-20	**0.024**	2.33E-40	0.001	6E-21	1.3E-19	**0.027**	8.8E-39	0.005
Copper						1.011	0.302	0.970	0.563	1.817	1.047	0.329	0.883	0.566	1.939
Faecal coliform											1.000	0.00043	0.955	0.999	1.001
Total coliform											1.000	9.6E-05	0.862	1.000	1.000

Note: *In bold are significant predictors.

In the second model, where heavy metals were accounted for, the odds of prediction for conductivity and magnesium remained less likely in predicting optimal water pH. Total dissolved solids, turbidity and total alkalinity were still significantly associated with predicting optimal pH in ground water systems. There was however partial mediation by the heavy metals (changes in significant values). It was observed that, total suspended solids was not statistically significant in model 1, but became statistically significant in model 2, indicating mediation by the heavy metals. In this instance, total suspended solids (OR = 0.009, p < 0.05) was associated with predicting optimal pH levels. Among the heavy metals, zinc (OR = 0.108, p < 0.05) and manganese (OR = 5.71*10–22, p < 0.05) were less likely to predict optimal pH for ground water systems.

In the third model in which biological parameters were accounted for, the model showed similar characteristics of model 2. Of the physicochemical parameters, the relationship between conductivity and magnesium and the likelihood of predicting non-optimal pH levels persisted in the third model. Total dissolved solids, total suspended solids, turbidity and total alkalinity remained significantly associated with higher odds of predicting optimal pH and their odds of prediction persisted as observed in model 2. Among the heavy metals, the relationship between zinc, manganese and the odds of predicting pH levels also remained robust and persisted. In this case zinc and manganese were associated with predicting odds of non-optimal water pH. None of the bacteriological factors were statistically significant in predicting pH levels in ground water systems in this study.

### Multivariate regression model showing the relationship between pH and selected water quality parameters for surface water

Multivariate regression model for surface water is shown in Table 6. Physicochemical parameters were accounted for in model 1. Model 2 accounted for heavy metals and the third model controlled for biological parameters. In the first model, turbidity (OR = 1.034, p < 0.05), calcium (OR = 1.055, p < 0.001) and sulphate (OR = 0.994, p < 0.05) showed statistically significant association with water pH levels. In this case, turbidity and calcium were more likely to predict optimal water pH levels. Contrariwise, sulphate was associated with non-optimal values of pH in surface water.

**Table 6:** Multivariate complementary log–log regression model predicting the relationship between pH and water quality parameters for surface water.

	SURFACE WATER														
	Physicochemical Model					Physicochemical parameters and Heavy Metal Model					Physicochemical, Heavy metal and Biological Model				
Variables	OR	Robust SE	P Value	Conf. Interval		OR	Robust SE	P Value	Conf. Interval		OR	Robust SE	P Value	Conf. Interval	
Conductivity	0.992	0.004	0.052	0.985	1.000	0.987	0.005	**0.018**	0.977	0.998	0.993	0.005	0.163	0.984	1.003
Total dissolved solids	1.009	0.006	0.148	0.997	1.022	1.010	0.007	0.183	0.995	1.025	1.001	0.007	0.878	0.988	1.014
Total suspended solids	1.009	0.007	0.208	0.995	1.023	1.018	0.008	**0.024**	1.002	1.035	1.028	0.017	0.096	0.995	1.063
Turbidity	1.034	0.017	**0.049**	1.000	1.068	1.023	0.012	**0.045**	1.000	1.047	1.116	0.039	**0.002**	1.043	1.195
Calcium	1.055	0.014	**0.000**	1.027	1.083	1.080	0.025	**0.001**	1.032	1.131	1.089	0.027	**0.001**	1.037	1.143
Magnesium	0.967	0.037	0.381	0.897	1.042	0.963	0.042	0.392	0.884	1.049	0.956	0.047	0.354	0.869	1.052
Sulphate	0.994	0.003	**0.029**	0.988	0.999	0.999	0.004	0.862	0.992	1.007	0.988	0.007	0.102	0.974	1.002
Nitrate	1.006	0.007	0.437	0.991	1.020	1.017	0.012	0.140	0.994	1.040	1.028	0.012	**0.019**	1.005	1.052
Phosphate	1.026	0.017	0.130	0.993	1.060	1.014	0.019	0.436	0.979	1.051	1.029	0.021	0.169	0.988	1.072
Total alkalinity	1.043	0.489	0.929	0.416	2.614	4.523	3.694	0.065	0.912	22.423	5.488	7.599	0.219	0.364	82.790
Arsenic						0.002	0.047	0.829	5.4E-29	4.63E+22	4.8E-32	1.9E-30	0.066	1.7E-65	135.304
Zinc						0.003	0.008	**0.031**	1.7E-05	0.583	0.002	0.007	0.124	5.2E-07	5.700
Iron						4.938	4.770	0.098	0.743	32.796	1.404	1.598	0.766	0.151	13.068
Manganese						168614.3	583503.2	**0.001**	191.092	1.49E+08	1.5E+07	7.9E+07	**0.002**	509.349	4E+11
Copper						0.593	0.123	**0.012**	0.394	0.892	0.569	0.165	0.052	0.322	1.004
Faecal coliform											0.985	0.003	**0.000**	0.979	0.991
Total coliform											1.005	0.002	**0.005**	1.002	1.009

Note: *In bold are significant predictors.

In the second model, where heavy metals were accounted for, only turbidity (OR = 1.023, p < 0.05) and calcium (OR = 1.083, p < 0.05) persisted in predicting water pH levels. However, new relationships appeared, indicating mediation by the heavy metals. Here, conductivity (OR = 0.987, p < 0.05) and total suspended solids (OR = 1.0180, p < 0.05) were statistically significant in predicting water pH levels. Conductivity in this scenario was less likely to predict optimum water pH levels while total suspended solids was more likely to predict optimal water pH. Of the heavy metals, manganese (OR = 168614.3, p < 0.05), zinc (OR = 0.003, p < 0.001) and copper (OR = 0.593, p < 0.05) were statistically associated with the odds of predicting water pH. In this instance, manganese was more likely to predict optimal water pH levels. Contrariwise, higher values of zinc and copper were associated with non-optimal levels of pH in surface water systems.

Total suspended solids and conductivity lost their significance in the third model when biological factors were accounted for. Indicating mediation by the biological factors. Turbidity (OR = 1.116, p < 0.05) and calcium (OR = 1.089, p < 0.05) remained robust and persisted in predicting pH levels. In this case, they were more likely to predict optimal water pH levels. A new relationship also appeared. Here, nitrate (OR = 1.028, p < 0.05) became statistically significant in predicting pH levels. Thus, higher values of nitrate was more likely to predict optimal pH levels. Of the heavy metals, manganese (OR = 1.5*107, p < 0.05) remained significant with high odds of predicting optimal pH in surface water locations. Among the bacteriological factors, total coliform (OR = 1.005, p < 0.05) and faecal coliform (OR = 0.985, p < 0.001) were statistically associated with predicting water pH levels. Total coliform in this scenario was associated with the odds of predicting optimal pH values while faecal coliform was associated with non-optimal pH values.

## Discussion

This study analyzed the association between pH and physicochemical parameters while controlling for heavy metals and bacteriological factors for ground water and surface water systems in the Tarkwa mining area. Nested logistic regression model was used to evaluate the dynamics of these relationships in groundwater and surface water systems. The chemistry of water systems, especially heavy metals, are much affected by pH and vice versa [27]. Knowledge of the association between water quality parameters is important for the sustainability and quality management of water. This study used heavy metals, bacteriological and physicochemical factors as predicting variables to assess the association between water quality parameters and pH and how their associations vary in the different water systems. The models in this study indicated that higher values of pH can be associated with some water quality parameters and can give an idea of the quality of water system.

In a zero-order relationship for the ground water system, six physicochemical parameters (conductivity, total dissolves solids, total suspended solids, total alkalinity, calcium and sulphate) were associated with predicting optimum water pH levels. High values of these parameters implied high probability of having water optimal for drinking.

At the multivariate level for ground water, total alkalinity was consistent with the odds of prediction. The addition of heavy metals and bacteriological factors further strengthened the relationship between alkalinity and odds of predicting optimal pH. The carbonate system is a function of alkalinity while the various forms of carbonates (carbon dioxide, bicarbonate and carbonates) govern pH conditions of water [28]. High alkaline water often has high pH and so the strong association of total alkalinity with optimal pH indicates that the alkalinity values reported in this study are not high and thus corresponds to non-optimal pH.

Total dissolved solids were robust in its association with pH. The major contributors of total dissolved solids are carbonates, bicarbonates and salts of sulphates, phosphates, chlorides and nitrates. The dissolution of these salts (sulphate and phosphate) influences the availability of dissolved solids, the presence of dissolved solids indicates the dissolution of salts. Tlili-Zrelli [29] observed a linear relationship between total dissolved solids and major ions in groundwater. Solids that dissolve in water break into positively and negatively charged ions thereby increasing the conducting ability of water [30]. Conductivity, a property that depends mainly on dissolved salts can be taken as indirect measure for total dissolve solids [31]. These dissolved ions consequently become the conductors for electric current. This linear relationship between conductivity and total dissolved solids was further manifested in the correlation analysis as a strong positive correlation was observed. The relationship between conductivity and dissolved solids indicates the degree to which salts dissociate into ions. Armah [32] reported a significant association between conductivity and total dissolved solids with the distribution of pH in a ground water system. Interestingly, in this study, increased total dissolved solids indicates optimal pH while increased conductivity indicates non-optimal water pH.

Magnesium was insignificant while sulphate and calcium were significantly associated with pH levels in the zero-order relationship, however, the opposite occurred in the multivariate model where other physicochemical parameters, heavy metals and bacteriological factors were accounted for. In all three models for groundwater, magnesium was significantly associated with lower odds of predicting optimum water pH with slight decrease in odds as heavy metals and biological factors were accounted for, indicating that higher values of magnesium is associated with non-optimal groundwater pH. The association of magnesium with pH at the multivariate level could be mediated by sulphate ions. Kura [33] in his study reported that sulphate and magnesium ions were significantly associated and could have influence each other in a complex system. Magnesium bearing minerals such as dolomite is a very common mineral in groundwater resulting from rock-water interactions. The dissolution of dolomite is a function of pH, moreover, the fractionation of carbonate rocks is also influenced by water pH [34]. Greiserman [35] also reported similar observation where the dissolution of dolomite into calcium and magnesium ions were effective in acidic medium. These studies are in line with this current study that, high concentration of magnesium ions in water suggest non-optimal pH and that the availability of magnesium in water especially in groundwater system is influenced by pH.

Turbidity was not significant in predicting pH levels in the zero-order relationship however, it became significantly associated with pH at the multivariate level for groundwater, with higher values of turbidity in this case indicating an optimal water pH. Suspended solids contribute to the turbidity of water and could be the main parameter that mediated the association between turbidity and pH. Many studies including Acheampong [36] and Mustapha [30] also observed a significant relationship between total suspended solids and turbidity. Interestingly, total suspended solids lost its significance with pH in the physicochemical model. However, when heavy metals and bacteriological factors were controlled for, the relationship between pH and total suspended solids reappeared, with higher values of total suspended solids indicating optimal water pH.

Among the heavy metals, only zinc and manganese were significantly associated with pH; predicting non-optimal pH levels. The environment of every chemical specie has influence on its behavior and thus affect its reactions with other species. Although solubility of metals depends on pH, the chemical composition of water systems can influence metal dissolution. The availability of metals in a groundwater system is a complex function of many factors including chemical, biological, and environmental processes [37]. For the bacteriological factors, both faecal and total coliform were significant predictors of pH in the zero-order analysis but was however insignificant in the multivariate analysis. Thus, the relationship between coliform bacteria and pH in a water system can be mediated by the physicochemical factors and heavy metals.

For the zero-order analysis in the surface water system, total suspended solids, turbidity, total alkalinity and calcium were all associated with optimal water pH. However, only turbidity and calcium were significantly associated with pH in the multivariate regression model. Sulphate also became significantly associated with pH.

Calcium showed a strong association with high odds of predicting pH, and was persistent in all three models, this result indicated that higher values of turbidity and calcium are associated with optimum water pH. The interaction between carbon dioxide and solid carbonates in the form of calcium carbonate (calcite) from bedrocks liberates calcium ions and bicarbonate species in water. Calcium is an important component of the carbonate system and its liberation from carbonate system is affected by pH. Holland [38] observed a linear relationship between calcium ions and bicarbonate ions in river waters. The association of calcium with pH in this study further suggest that pH affect the carbonate system. The dissolution of metals contribute to dissolved ions and thus the introduction of heavy metals influenced the availability of dissolved ions and consequently mediating the association of conductivity with pH.

Total alkalinity and total suspended solids were significantly associated with high odds of prediction in the zero-order analysis but lost their significance in the physicochemical model of the multivariate analysis for surface water. However, the addition of heavy metals mediated the association of total suspended solids with surface water pH. Insoluble metal ions in the form of solid elemental metal precipitates (metal colloids) and solid metal compounds might have contributed suspended solids. The nature of surface water makes it susceptible to receiving solids in all forms through runoffs, agricultural inputs, waste water discharge, etc. and could also contribute to solid particles.

All metals in the zero-order analysis for surface water had no significant association with pH levels. However, zinc, manganese and copper were significantly associated with pH at the multivariate level with manganese values associated with high odds of predicting optimum water pH. Zinc and copper were however not robust in the multivariate model accounting for biological and physicochemical parameters. The nature of surface water allows it to be much affected by climatic conditions; the physical conditions of surface water such as temperature, turbulence and transparency influence its chemical and biological process. Temperature is known to affect mobility and solubility of chemical species while turbulence affect turbidity via water overturn (mixing) and consequently affecting water temperature. The variations in physical conditions of surface water subject water parameters to constant changes and could account for the non-robustness of some surface water variables.

When biological factors were accounted for in the surface water model, both faecal and total coliforms showed significant association with pH. Interestingly, total coliforms were associated with optimal pH values while faecal coliform were associated with non-optimal water pH, a similar observation was reported by Aram et al. [39]. This further implied that total coliform bacteria survival is much favored in optimal pH conditions. The distribution of feacal bacteria in water are much affected by physical and climatic factors such as runoffs, temperature and solar radiations than pH. Both total and faecal coliform bacteria are considered pollution indication bacteria and are used as a measure for sanitary parameters for water in a particular environment. Several studies have also reported a significant relationship between various bacteria in water environment [40–43]. The introduction of biological factors also saw nitrate gaining significant association with pH. The association of nitrate with pH suggests that an increase in the nitrogenous species occur in optimal pH levels. Armah et al. [44] reported a significant association between nitrate and coliform bacteria and thus nitrate’s significance in the biological model could be influenced by the introduction of coliform bacteria. The variations in chemical composition of surface water are highly influenced by topography, climate, and mineralogical composition of the bed rock [45]. Empirical research has shown that the quality of surface water in a particular region is controlled by these natural factors [46]. Surface water is much affected by temperature and could be an influential factor in the availability of both faecal and total coliform [47] and consequently their association with water pH.

## Conclusion

This study sough to evaluate the relationship between water pH and physicochemical properties of water while controlling for the effect of heavy metals and bacteriological factors using a nested logistic regression model. The study also compared the relationship between water quality parameters in confined water systems (ground water) and open water systems (surface water). The findings of this study give the joint effect of water quality parameters on pH and how they affect each other in a confined and open water system. For the zero order relationship in groundwater, EC, TDS, TSS, Ca, SO_4_^2-^, total alkalinity, Zn, Mn, Cu, faecal and total coliform were more likely to predict optimal water pH. For surface water however, only TSS, turbidity, total alkalinity and Ca were significant predictors of optimal pH levels. At the multivariate level for groundwater, TDS, turbidity, total alkalinity and TSS were associated with optimal water pH while EC, Mg, Mn and Zn were associated with non-optimal water pH. For surface water multivariate regression model, turbidity, Ca, TSS, NO_3_, Mn and total coliform were associated with optimal pH while SO_4_^2-^, EC, Zn, Cu, and faecal coliform were associated with non-optimal water pH. The non-robustness of predictors in the surface water models were conspicuous. The results indicate that the relationship between water pH and other water quality parameters are different in different water systems and can be influenced by the presence of other parameters. Associations between the parameters are steadier in groundwater systems due to its confined nature. Extraneous inputs and physical variations subject surface water to constant variations which reflected in the non-robustness of predictors. The carbonate system was influential in how water quality parameters associate with one another in both ground and surface water systems. This study affirms that chemical constituents in natural water bodies react in a more complicated ways than if they were isolated and that the interaction between various parameters could predict the quality of water in a particular system.
